# Flowering time and the identification of floral marker genes in *Solanum tuberosum* ssp. *andigena*

**DOI:** 10.1093/jxb/erz484

**Published:** 2019-10-28

**Authors:** Tanja Seibert, Christin Abel, Vanessa Wahl

**Affiliations:** 1 Max Planck Institute of Molecular Plant Physiology, Department of Metabolic Networks, Am Mühlenberg, Potsdam, Germany; 2 University of Nottingham, UK

**Keywords:** Flowering time, marker genes, potato, *Solanum tuberosum* ssp. *andigena*, *StAN*, *StFD*, *StLFY*, *StMC*, *StSOC1*, *StWOX9*

## Abstract

Solanaceae is a family of flowering plants that includes agricultural species such as tomato (*Solanum lycopersicum*), eggplant (*S. melongena*), pepper (*Capsicum annuum*), and potato (*S. tuberosum*). The transition from the vegetative to reproductive stage has been extensively investigated in tomato as it affects fruit yield. While potato has mainly been studied with regards to the formation of storage organs, control of flowering time is a subject of increasing interest as development of true seeds is becoming more important for future breeding strategies. Here, we describe a robust growth regime for synchronized development of *S. tuberosum* ssp. *andigena*. Using SEM to analyse the developmental stages of the shoot apical meristem (SAM) throughout the floral transition, we show that *andigena* is a facultative long-day plant with respect to flowering. In addition, we identify the flower meristem identity gene *MACROCALYX* (*StMC*) as a marker to distinguish between the vegetative and reproductive stages. We show that the expression of *WUSCHEL HOMEOBOX 9* (*StWOX9*) and *ANANTHA* (*StAN*) are specific to the inflorescence meristem and flower meristems in the cyme, respectively. The expression patterns of homologs of Arabidopsis flowering-time regulators were studied, and indicated that *SUPPRESSOR OF OVEREXPRESSION OF CONSTANS1* (*StSOC1*) and *StFD* might regulate flowering similar to other plant species.

## Teaser

*MACROCALYX*, *WUSCHEL HOMEOBOX9*, *ANANTHA*, *SUPPRESSOR OF OVEREXPRESSION OF CONSTANS1*, and *FD* are expressed in distinct domains in the shoot apex in *Solanum tuberosum* ssp. *andigena* and provide good markers to distinguish developmental stages and organs.

## Introduction

In potato (*Solanum tuberosum*), developmental transitions are influenced by external cues such as temperature, soil composition, day length, and light, as well as internal cues such as the carbohydrate status, age, and plant hormones (Rodríguez-Falcón *et al*., 2006). The induction of tuberization is strongly dependent on a critical day length, which varies between potato genotypes (Ewing, 1978). In wild Andean genotypes, such as *S. tuberosum* ssp. *andigena*, tuber formation is strictly dependent on short days (SD) or, rather, long nights (Abelenda *et al.*, 2014). Modern cultivars have been extensively bred against this sensitivity to photoperiod; however, tuberization remains largely promoted by reduced day length and cold temperatures in order to ensure survival during cool winter periods (Abelenda *et al.*, 2011).

Potato plants produce berries that contain 100–400 seeds. Due to their heterogenic nature, these seeds produce offspring that are genetically different from their mother plant, and hence propagation of genetically identical plants from seeds for agricultural use is not currently possible. To overcome variation in plant performance and yield, conventional potato cultivation has adopted a clonal propagation strategy, by making use of tuber pieces, or so-called ‘seed potatoes’. Recent cultivation approaches are making use of true potato seeds that are genetically identical due to haploidization and inbreeding of the parental plants (Lindhout *et al.*, 2018). True potato seeds will certainly become more important in the future, as they are free of pathogens and easier to transport compared to seed potatoes, thereby improving agricultural logistics. In addition, millions of true seeds can be bulked and distributed during a single season, using grams of seeds instead of kilograms of seed potatoes. Improving our understanding of the genetic regulatory networks that underlie flowering and tuberization in potato is crucial not only for further crop improvement, but also for optimizing the production of true potato seeds.

For the survival of a plant species to be ensured, the transition from vegetative to reproductive growth (formation of viable seeds or tubers) needs to be tightly controlled. Flowering time has been extensively studied in the model plant species Arabidopsis, a facultative long-day (LD) plant. Flowering time in *S. tuberosum* might be independent of the photoperiod, similar to day-neutral flowering in tomato (*S. lycopersicum*) (Molinero-Rosales *et al.*, 2004; Lifschitz and Eshed, 2006; Navarro *et al.*, 2011); however, there remains some ambiguity about the influence of photoperiod on flowering time in potato, as most studies have been carried out in LDs and very little information on SD conditions is available.

Upon floral induction, the vegetative shoot apical meristem (SAM) changes into an inflorescence meristem (IM), giving rise to flower meristems (FMs) that produce flowers, fruits, and seeds. The IM is maintained in Arabidopsis, leading to indeterminate growth of an inflorescence (Shannon and Meeks-Wagner, 1991; Alvarez *et al.*, 1992; Bradley *et al.*, 1997). In potato, the transition from a vegetative to a reproductive SAM is accompanied by temporal and spatial changes of growth and cell divisions at the SAM, and concomitant morphological changes. Within the vegetative SAM, the central zone is the slowest growing region, while mitotic activity is strongly increased during the floral transition (Kwiatkowska, 2008). This is reflected by a change from a flat meristem towards a dome-shaped meristem (DM), which indicates the high rate of cell divisions at the early transition stage, followed by the initiation of two determinate IMs through cleavage of the SAM (Danert, 1957). These meristems undergo further cleavages, each producing an FM and an IM, resulting in the formation of a double-scorpioid cyme. Some species are capable of producing four scorpioid cymes by additional cleavages of the two IMs, giving rise to four independent IMs (Hart and Hannapel, 2002).

In Arabidopsis, flowering is induced by a wide range of environmental and endogenous signals that are integrated in the leaves and the SAM to induce flowering (Srikanth and Schmid, 2011). In potato, both the transition to flowering and the induction of tuberization are dependent on mobile proteins referred to as ‘florigen’ and ‘tuberigen’, respectively. Translocation to their signal perception sites and the concomitant induction of target genes is crucial to induce the developmental transition. *FLOWERING LOCUS T* (*FT*), which is expressed in phloem companion cells of leaves, induces flowering in Arabidopsis. FT moves to the SAM to interact with the bZIP transcription factor FD to induce flowering (Corbesier *et al.*, 2007; Jaeger and Wigge, 2007; Mathieu *et al.*, 2007). In tomato and rice (*Oryza sativa*), FT binds to FD and a 14-3-3 protein, forming a so-called ‘flowering activation complex’ (FAC), which regulates the expression of downstream targets (Pnueli *et al.*, 2001; Taoka *et al.*, 2011). Together, they activate flower meristem identity genes such as *SUPPRESSOR OF OVEREXPRESSION OF CONSTANS1* (*SOC1*), *APETALA1* (*AP1*), and *FRUITFUL* (*FUL*) (Abe *et al.*, 2005; Wigge *et al.*, 2005). In Arabidopsis, AP1 in turn modulates the expression of a key meristem identity gene, *LEAFY* (*LFY*), which is a conserved plant-specific transcription factor that activates floral homeotic genes (Weigel *et al.*, 1992; Blázquez *et al*., 1997). In potato, two genes encoding homologs of *FT* regulate either flowering or tuberization: StSP3D is considered the major player in the regulation of flowering while StSP6A is the tuberigen (Navarro *et al.*, 2011). *StSP6A* is expressed in the leaves and its protein product translocates to underground stems, so-called ‘stolons’, to induce tuberization (Navarro *et al.*, 2011). It has been shown that StSP6A interacts with St14-3-3 proteins, StFD, and StFD-like (FDL) (Teo *et al.*, 2017). Work by Teo and colleagues suggests that a TUBERIGEN ACTIVATION COMPLEX (TAC) is present in stolon tips of *S. tuberosum* (Teo *et al.*, 2017), similar to the FAC at the SAM (Pnueli *et al.*, 2001; Taoka *et al.*, 2011). The downstream targets of the TAC complex are unknown and it is unclear whether a similar FAC is involved in the regulation of flowering time at the SAM in *S. tuberosum*. Direct downstream targets of *StSP6A* to induce tuberization in below-ground stolons are unknown. However, an ethanol-inducible system to activate *StSP6A* expression has been shown to up-regulate tuber-specific genes (e.g. *StGA2ox1*) in stolons to similar levels as those observed in swelling stolons in wild-type plants (Navarro *et al.*, 2011). Interestingly, flowering pathways investigated mainly in Arabidopsis (Blümel *et al.*, 2015; Bouché *et al*., 2015) and rice (Tsuji *et al.*, 2011) have been found to share many components with tuberization-inducing pathways in potato (Rodríguez-Falcón *et al*., 2006).

In this study, we provide a detailed analysis of developmental transitions in potato in terms of morphological features and the molecular players involved. We used *S. tuberosum* ssp. *andigena* to investigate flowering independent of tuber formation, as it is a strict SD-dependent variety in which tuberization is only induced when plants are transferred to SD conditions. Thus, flowering time can be studied under LD conditions without an effect of tuber-inducing signals. We developed a stable growth protocol to give the most uniform growth of plants for both *in vitro* culture and cultivation in soil to study flowering time and tuberization. We describe the morphological changes that occur at the SAM during flowering in potato as shown by SEM. Using RNA *in situ* hybridization in shoot apices during the floral transition, we identify *MACROCALYX* (*StMC*) as a floral marker gene that is expressed in distinct domains in the SAM upon floral induction. Finally, we show that two *StSOC1* homologs and two *StFD-like* transcripts are expressed in the SAM during flowering.

## Materials and methods

### Plant material and growth conditions

The wild potato species *Solanum tuberosum* ssp. *andigena* (line 7540; Navarro *et al.*, 2011), was used for this study. *In vitro* propagation of plants, node sections, and head cuttings were prepared under sterile conditions, and grown on Murashige and Skoog (MS) medium (Murashige and Skoog, 1962) containing 2 % (w/v) sucrose, supplemented with B-vitamins (Duchefa Biochemie, Haarlem, Netherlands). Plants in tissue culture were grown in a low-light growth chamber (50–75 µmol m^–2^ s^–1^, 22 °C) under LD conditions. After transfer of the plantlets to soil, they were moved to a high-light growth chamber and grown at 22 °C and 60% humidity with a light intensity of 300–400 µmol m^–2^ s^–1^ (metal halide lamps, MT400DL/BH 400W, Iwasaki Electric Co Ltd, Tokyo, Japan) under either LD conditions (16 h light, 8 h dark) or SD conditions (8 h light, 16 h dark), depending on the experiment. For tuberization experiments, the plants were grown in LD conditions for 27–30 d and were then transferred to SD conditions (Supplementary data S1, Fig. S1 at *JXB* online). All plants were grown in standard soil consisting of a peat and sand mixture (2:1) (N, 160–300 mg l^–1^; P_2_O_2_, 160–300 mg l^–1^; K_2_O, 240–330 mg l^–1^). During the first week in soil, young plants were protected from high irradiance using a light-shading cloth. Plants were first grown in small pots (6.5×6.5 cm) before being transplanted to bigger pots (11×11 cm, height 21 cm, 2 l) at 14 days after transfer (DAT) to soil.

### Flowering time analysis

The flowering time of *S. tuberosum* ssp. *andigena* was determined based on a morphological analysis of the SAM. Using fine forceps and a scalpel, the SAM was uncovered by removing the leaves and young primordia surrounding the apex. Indicative changes in morphology that occur throughout the floral transition were used to determine the individual development stages in the SAM. This was carried out using either a MZ FIII stereo-microscope (Leica) or a Hitachi Tabletop Microscope TM3030Plus. To determine the flowering percentage of plants, SAMs displaying the initial cleavage (C1) stage, as well as older developmental stages (C2, young flowers, etc.), were considered as flowering.

### SEM analysis

Samples were taken from plants grown under LD conditions. Older leaves surrounding the shoot apex were removed under the stereo-microscope until the SAM was exposed. Samples were frozen in liquid nitrogen and mounted on steel sample stubs prior to image acquisition using the Hitachi Tabletop Microscope (observation mode, SE image; observation conditions, 5–15 kV).

### RNA *in situ* hybridization

RNA *in situ* hybridization was performed as described by Wahl *et al.* (2013). Briefly, tissue samples were taken at end of the day (ED) and were immediately transferred into freshly prepared FAA fixative (formaldehyde–acetic acid–ethanol), and apices were isolated by removing all but the smallest leaves surrounding the meristem. The samples were subsequently dehydrated with ethanol and infiltrated with paraffin wax (Paraplast, Leica) using an automated vacuum-embedding system (ASP300S, Leica). After infiltration, the apex samples were immediately processed in an embedding centre (EG1160, Leica). Using a rotary microtome (RM2265, Leica), 8-µm sections were made and transferred to polysine-coated slides (Roth, Karlsruhe, Germany).

Probes for full-length *StAN*, *StWOX9*, *StLFY*, *StMC*, *StSOC1*, *StSOC1-like*, *StFD*, and *StFD-like* were generated from their cDNAs, cloned into the pGEM^®^-T Easy Vector (Promega), and synthesized with a DIG RNA Labeling Kit (Roche). Gene identifiers, oligo sequences, and protein alignments are provided in Supplementary Table S1, Figs S4–S9.

The sections were imaged using an Olympus BX-61 microscope and the cellSens Dimension software (Olympus). Single sections from apices were imaged as a series of multiple pictures covering the section, which were later aligned to display the entire tissue section using Photoshop CS6 (Adobe). For each gene, the non-complementary sense probes were used as negative controls (Supplementary Fig. S10).

## Results

### Optimized growth protocol to study flowering time

To study the molecular mechanisms regulating the timing of the transition to flowering, we first developed an optimal system to grow plants in order to guarantee reproducible results in all future experiments. The standardized growth protocol comprised a stringent regime of tissue culture and growth in soil. For *in vitro* cultivation, three steps were essential for uniform plant growth. (i) Initially, main plant shoot tips of ~2 cm, containing 3–4 young leaves, were transferred to new medium under sterile conditions and grown for 4 weeks. (ii) Before side shoots grew out from axillary meristems (AMs), node cuttings containing an axillary bud were transferred to new medium in such a way that the cut stem sides were submerged in the medium with the axillary bud facing upwards. These explants were cultivated for 16 d. During this time, lateral shoots emerged from the AMs (2–3 cm). (iii) These new shoots were cut and transferred to new medium for root formation. During the entire tissue culture period, plants were grown in a walk-in climate chamber in LD conditions (16 h light, 8 h dark) at a constant temperature of 22 °C, with light intensities between 50–75 µmol m^–2^ s^–1^.

**Fig. 1.**
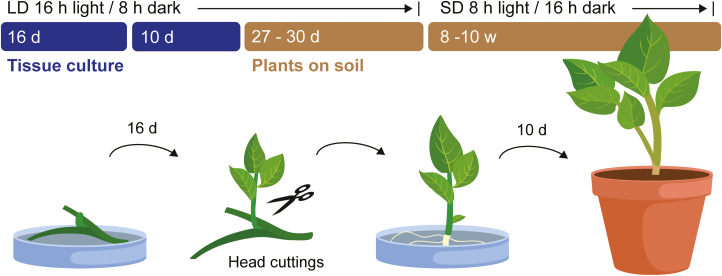
Experimental growth regime and growth system developed for *Solanum tuberosum* ssp. *andigena*. Node sections were cultured on MS medium for 16 d until side shoots grew out. These shoots were then cut and transferred to new medium to induce root growth. The tissue culture conditions were long days (LD, 16 h light/8 h dark), constant 22 °C, 50–75 µmol m^–2^ s^–1^. After 10 d, the plants were transferred to soil and grown in high-light chambers at constant 22 °C, 60% humidity, 300–400 µmol m^–2^ s^–1^ in LD conditions for 27–30 d. At 14 d after transfer to soil, plants were transferred to bigger pots. For tuberization experiments and for quantification of tuber yield, plants were transferred to short days (SD, 8 h light/16 h dark) and grown for another 8–10 weeks (w).

When roots were fully developed (typically after 10 d), the plants were transferred to soil. A mixture of peat and sand containing all the necessary nutrients for normal potato growth was used, as described in the Methods. Finally, plants in soil were transferred to a growth chamber at a constant temperature of 22 °C, 60% humidity, and 300–400 µmol m^–2^ s^–1^ in LD conditions (flowering time experiments). The plants remained under LDs for 27–30 days after transfer (DAT) to soil before they were transferred to SD conditions for tuberization experiments (Fig. 1), for which they were first grown in small pots and transferred into bigger pots after 14 DAT (Supplementary Fig. S1). This stringent protocol ensured uniformly growing plants with little variation in overall appearance and development (Supplementary Fig. S2).

### Morphological changes associated with flowering time

**Fig. 2.**
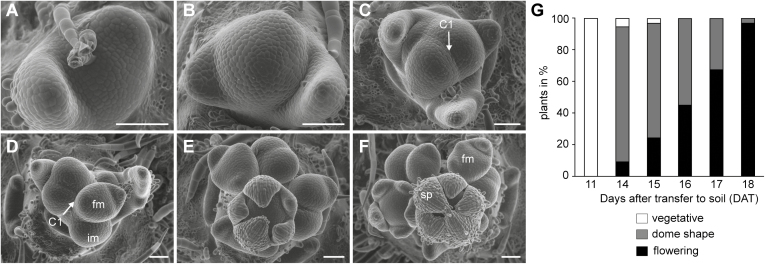
Morphological changes at the shoot apical meristem (SAM) during the floral transition in *Solanum tuberosum* ssp. *andigena*. (A) Vegetative shoot apex 11 d after transfer (DAT) to soil. (B) At 15 DAT the SAM size increases and this is followed by (C) the initial cleavage (C1) of the meristem at 16 DAT to produce two determinate inflorescence meristems (IM). (D) At 17 DAT, the initial cleavage is more pronounced and both IMs have undergone an additional cleavage, each resulting in a flower meristem (FM) and an IM. Maturing inflorescences at (E) 18 DAT and (F) 19 DAT. Mature flowers with sepals (sp), petals, and stamens (covered by sepals) are formed from the FMs. Representative images are shown from *n*=5 replicates. Scale bars are 100µm. (G) Analysis of the timing of the floral transition in *andigena* wild-type plants grown under long-day (LD) conditions (16/8 h light/dark), scored using a stereo-microscope. The SAM was made accessible by removing leaves and leaf primordia. All plants (100%) were vegetative at 11 DAT. After 14 DAT, 85.7% of the plants were observed to be dome-shaped, while 5.4% and 8.9% were vegetative and flowering, respectively. Almost all plants were flowering after 18 DAT. Total number of SAMs examined: 11 DAT, *n*=100; 14 DAT, *n*=56; 15 DAT, *n*=61; 16 DAT, *n*=87; 17 DAT, *n*=95; 18 DAT, *n*=32; 19 DAT, *n*=30; 20 DAT, *n*=28.

SEM was used to study the morphological changes at the SAM of dissected apices, from which leaves had been removed. Plants were grown as described above in LD conditions, and apices were dissected in a dense time series starting at 11 DAT and every day between 14–19 DAT. Using this approach, we determined that the time-frame during which the floral transition occurred under our growth conditions was between 15 DAT and 16 DAT (Fig. 2). At 11 DAT, plants were in the vegetative phase (Fig. 2A). At ~15 DAT, the size of the SAM significantly increased, leading to the formation of a DM (Fig. 2B). This increase in size marked the first morphological change that indicated the transition from a vegetative to a reproductive SAM. This stage was followed by the generation of the reproductive SAM through the first cleavage (C1), giving rise to two determinate IMs around 16 DAT (Fig. 2C). Both these IMs underwent several consecutive cleavage events (C2, C3, etc.), each time resulting in a FM and an IM, respectively, with one of the IMs progressing slightly faster than the other (Fig. 2D–F). After 3 weeks in soil, mature flowers developed from the FMs and were organized in two independent whorls.

For more detailed analysis of morphogenesis at the SAM, a larger number of plants (*n*=460) was visually examined under a stereo-microscope. The results reproduced the timing of the observed developmental changes described above. At 11 DAT, 100% of the SAMs were still vegetative. After 14 DAT, 85.7% of the plants displayed an increased SAM size (dome shape), while only 5.4% displayed a vegetative SAM. At the same time, a small number of plants (8.9%) showed signs of the initial cleavage and were hence considered to be flowering. The number of flowering plants increased over the following days and almost all plants had flowered after 18 DAT (96.77%). Previous research on flowering time in potato has mostly monitored the appearance of flower buds and anthesis (Chincinska *et al.*, 2008; Martin *et al.*, 2009; González-Schain *et al*., 2012; Plantenga *et al*., 2018). However, under our conditions, flower buds were visible by eye only after 4 weeks, which was 10–14 d after the floral transition at the SAM.

In Arabidopsis, flowering time can be measured either by the days to bolting or by the total leaf number, which correlates with the time to transition from a vegetative to a reproductive meristem (Koornneef *et al.*, 1991; Weigel and Glazebrook, 2002). We therefore counted the total number of visible leaves of potato plants at a single time-point (17 DAT) in several independent experiments in LD conditions. In contrast to Arabidopsis, we could not find a significant correlation between the total leaf number and the morphological stage of the SAM.

*Solanum tuberosum* ssp. *andigena* and other potato varieties have been assumed to initiate flowering independently of the day length, similar to what has been shown for tomato (Ben-Naim *et al.*, 2006; Navarro *et al.*, 2011). To test this assumption, we examined flowering time in SD conditions by determining morphological changes at the SAM. Plants were grown in tissue culture using the protocol described above, but were moved directly to SD conditions after transfer to soil. We found that the flowering time was significantly delayed compared to LD conditions (Supplementary Fig. S3). At 30 DAT, which was the time when all plants were flowering in LD conditions, the SAM was still vegetative in SD. At 40 DAT most of the plants showed the typical dome-shape structure, indicating the onset of the transition to flowering, and 10 d later all plants were flowering. Thus, our results showed that *S. tuberosum* ssp. *andigena* plants could flower under both LD and SD conditions; however, flowering time was significantly delayed when the days were shorter. Our results also indicated that the signal to induce flowering was not readily produced in tissue culture (LD), which would, in theory, lead to similar flowering times in all photoperiods.

### Marker genes for the floral transition and flower organ development

**Fig. 3.**
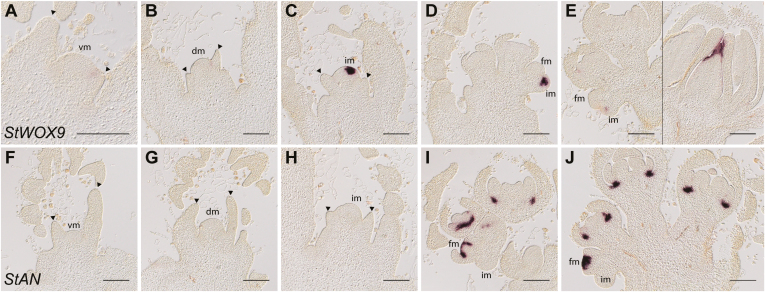
Expression of *StWOX9* and *StAN* during the development from vegetative to reproductive apices in *Solanum tuberosum* ssp. *andigena*. (A–E) *StWOX9* expression was detected at weak levels in the vegetative stage in the borders of the meristem, and was highly up-regulated in the inflorescence meristem (IM) during and following the floral transition. (F–J) In contrast, *StAN* expression was restricted to the reproductive stage and was detected in FMs and the flower anlagen. vm, vegetative meristem; dm, dome-shaped meristem; fm, flower meristem. Arrowheads indicate leaf primordia. Scale bars are 100 µm.

As shown using SEM, the potato inflorescence apex is a complex, three-dimensional structure (Fig. 2D–F). In addition to morphological analyses, genetic markers that are differentially expressed between the vegetative and reproductive stage are useful tools to discriminate flowering times of different cultivars, varieties, and transgenic lines. To better distinguish between the IM and FM, we analysed the spatial expression of *ANANTHA* (*AN*), a gene encoding an F-box protein that has been shown to be exclusively expressed in flowers to control the identity of flower organs in tomato (Lippman *et al.*, 2008). In parallel, we analysed the expression of *WUSCHEL HOMEOBOX 9* (*StWOX9*). WOX proteins are plant-specific transcription factors and homologs of the meristem maintenance protein WUSCHEL (WUS) (Haecker *et al.*, 2004). Among the 14 *WOX* genes in Arabidopsis, *AtSTIMPY*, which regulates embryonic patterning and meristem maintenance (Wu *et al.*, 2007), is most similar to *StWOX9*. In tomato, *SlWOX9* is specifically expressed in the IM, controlling the inflorescence architecture together with *SlAN* (Lippman *et al.*, 2008). We identified the paralogs of both genes in potato, hereafter referred to as *StAN* and *StWOX9*. For spatial expression analysis using RNA *in situ* hybridization, apices of wild-type *andigena* plants were harvested, fixed, embedded, and sectioned at 11, 14, 17, 20, and 23 DAT, covering vegetative to flowering stages. *StWOX9* was only weakly expressed in vegetative apices in the flanks of newly arising leaf primordia (Fig. 3A, B); however, it was strongly up-regulated upon the induction of flowering at the time-point of the first cleavage, which divides the SAM into an IM and FM. After the induction to flowering, *StWOX9* only remained strongly expressed in the IM (Fig. 3E). During flower development, we detected weak *StWOX9* signals, localizing between sepal and petal primordia. A similar pattern has been described for *SlWOX9* in tomato (Lippman *et al.*, 2008). We also identified *StWOX9* transcripts localized to the pistil of mature flowers.

In contrast to *StWOX9*, *StAN* was not expressed in the SAM during the vegetative stage or at the transition to flowering. Its expression was initiated only in FMs, and never overlapped with *StWOX9* expression. Expression in FMs was initially observed in a relatively broad domain of the central zone of the meristem, and it then reduced to the first cell layers excluding developing flower organs (Fig. 3, compare FM in I and youngest FM in J). *StAN* remained expressed in a subset of cells at the base of stamen filaments. In summary, both *StAN* and *StWOX9* showed similar expression domains compared to those reported in tomato (Lippman *et al.*, 2008).

**Fig. 4.**
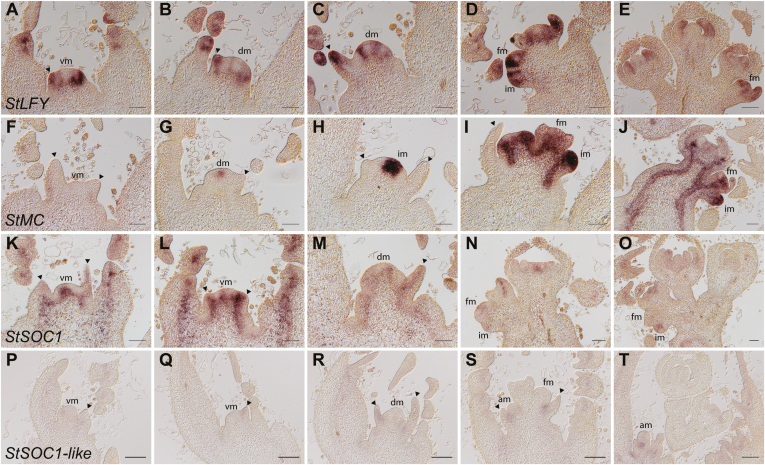
RNA *in situ* hybridization of the putative flowering time genes *StLFY*, *StMC*, *StSOC1*, and *StSOC1-like* in *Solanum tuberosum* ssp. *andigena*. Hybridization was done using longitudinal sections of the shoot apex of wild-type plants grown in long-day conditions (16/8 h light/dark), harvested at the end of the light period at 11, 14, 17, 20, and 23 d after the transfer of the plants to soil. Expression of (A–E) *StLFY*, (F–J) *StMC*, (K–O) *StSOC1*, and (P–T) *StSOC1-like* during the floral transition. vm, vegetative meristem; dm, dome-shaped meristem; im, inflorescence meristem; fm, flower meristem; am, axillary meristem. Arrowheads indicate leaf primordia. Scale bars are 100 µm.

To identify additional marker genes for the vegetative and reproductive stages of potato apices, we studied expression patterns of homologous floral integrator genes that have been identified in other plant species such as Arabidopsis, namely *LFY* (*LEAFY*), the flower meristem identity gene *AP1* (*APETALA1*), and *SOC1*. Our sequence analyses identified unambiguous homologs for *AP1* and *LFY*, and two putative candidates for the MADS-box gene *SOC1*. We named these genes according to their previously investigated homologs: *StLFY*, *StMC*, *StSOC1*, and *StSOC1-like*. *LFY* encodes a plant-specific transcription factor that has been shown to play key roles during flower development in diverse plant species (Weigel *et al.*, 1992; Weigel and Nilsson, 1995; Blázquez *et al*., 1997; Benlloch *et al.*, 2007; Moyroud *et al.*, 2010). *LFY* is weakly expressed in Arabidopsis leaves prior to the floral transition, but is induced strongly in FMs at later stages (Blázquez *et al*., 1997). In potato, we found signals indicating strong expression of *StLFY* in tissues other than FMs. In contrast to what is known in Arabidopsis, *StLFY* was expressed at the shoot apex throughout development, as shown by signals in leaf primordia and leaflets in young stages, when the SAM was still vegetative (Fig. 4A). After the transition to flowering, when the initial cleavage of the SAM into two IMs occurred, *StLFY* signals were found in young developing tissues, such as sepal primordia (Fig. 4D, E). Interestingly, signals were not observed in the presumptive cleavage site between the future IM and developing flowers in DMs (Fig. 4D). In flowers (after 23 DAT), strong signals were detected in tissues undergoing excessive growth, such as young flower organs. Overall, *StLFY* expression seemed strongest in proliferating cells of growing tissues, indicating functions other than floral induction during growth and development in potato.

*AP1* is a transcription factor responsible for flower meristem identity in Arabidopsis, and is required for the transition of an IM to an FM (Irish and Sussex, 1990; Mandel *et al.*, 1992; Bowman *et al.*, 1993). *AtAP1* is specifically expressed during the transition to flowering in primordia that are committed to a floral fate (Mandel *et al.*, 1992; Mandel and Yanofsky, 1995; Blázquez *et al*., 1997; Schmid *et al.*, 2005). In other plant species, orthologs of *AP1* have been shown to be conserved regulators of the floral transition (Distelfeld *et al.*, 2009; Pabón-Mora *et al*., 2012; Monniaux *et al.*, 2017; Wu *et al.*, 2017). In *S. lycopersicum*, a close relative of potato with a similar determinate growth habit, the *AP1* ortholog *SlMC* regulates inflorescence determinacy, sepal development, and other processes in reproductive development, such as fruit abscission (Vrebalov *et al.*, 2002; Nakano *et al.*, 2012). The closest ortholog to *AtAP1* and *SlMC* in *S. tuberosum* is *StMC*, which has not yet been functionally characterized. We cloned *StMC* of *S. tuberosum* ssp. *andigena* and used it as a probe for RNA *in situ* hybridization on a developmental series spanning the floral transition (Fig. 4F–J). Interestingly, *StMC* expression was not observed at the SAM during the vegetative stage. However, shortly before the first cleavage (14 DAT), *StMC* was weakly expressed in a central domain corresponding to the faster-developing IM next to the initial cleavage site. *StMC* expression strongly increased in the IMs upon the induction of flowering (Fig. 4H). After the floral transition, signals indicating *StMC* expression were observed in the IM, in vascular tissue, and in young flower organ primordia (sepals, petals, and carpels; Fig. 4I, J).

While *StLFY* and *StMC* showed a very distinct and specific pattern restricted to specific areas, such as organ boundaries, leaf primordia, and the IM in the shoot apex, the expression of *StSOC1* and its close paralog *StSOC1-like* were additionally found in the vasculature of older leaves (Fig. 4K–O, P–T). In Arabidopsis, *AtSOC1* is a key regulator of the floral transition that integrates signals from various flowering-time pathways such as the photoperiod, ambient temperature, vernalization, and gibberellic acid pathways (Borner *et al.*, 2000; Lee *et al.*, 2000; Samach *et al.*, 2000). *AtSOC1* is expressed in the vegetative stage in the vasculature of young leaves, but it is not expressed in the vegetative SAM (Samach *et al.*, 2000; Torti *et al.*, 2012). After the transition to flowering, *AtSOC1* is highly induced in the SAM. In contrast to Arabidopsis, we found that *StSOC1* was highly expressed in the centre of the meristem, and in the vascular tissue of leaf primordia and more mature leaves during the vegetative stage (11 DAT; Fig. 4K). After 14 DAT, *StSOC1* expression was first slightly up-regulated but it later decreased in the SAM and the leaf vasculature throughout the transition to flowering. It remained expressed in the IM and FMs at later stages (Fig. 4N, O). In addition to the meristematic expression, weak signals were observed in primordia of petals, sepals, and stamens, suggesting an additional role of *StSOC1* in the regulation of organ identity, as has been proposed for *AtSOC1* in Arabidopsis (Samach *et al.*, 2000). Overall, the expression pattern of *StSOC1* differed substantially from its Arabidopsis ortholog; however, its expression in the meristem might still support a role as a floral integrator gene. In contrast to *StSOC1*, the transcript abundance of *StSOC1-like* was much weaker and was mainly expressed in the vasculature of leaf primordia and leaves (Fig. 4P–T). Signals indicating *StSOC1-like* expression were never observed in the SAM or any lateral meristem throughout the transition to flowering.

**Fig. 5.**
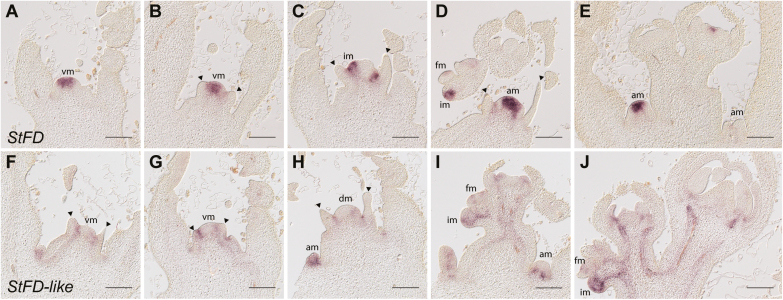
Expression of *StFD* and *StFD*-*like* on longitudinal sections of apices spanning the floral transition of wild-type *Solanum tuberosum* ssp. *andigena*. Hybridization was done using longitudinal sections of the shoot apex of plants grown in long-day conditions (16/8 h light/dark), harvested at the end of the light period at 11, 14, 17, 20, and 23 d after the transfer of the plants to soil. Expression of (A–E) *StFD* and (F–J) *StFD-like* during the floral transition. vm, vegetative meristem; dm, dome-shaped meristem; im, inflorescence meristem; fm, flower meristem; am, axillary meristem. Arrowheads indicate leaf primordia. Scale bars are 100 µm.

Upstream of the floral integrators, other factors have been identified to transduce exogenous and endogenous information via canonical pathways. One of these key players is *FT*, which promotes flowering through the activation of *SOC1* expression by the formation of a protein complex with the transcription factor FD in Arabidopsis (Abe *et al.*, 2005; Wigge *et al.*, 2005; Yoo *et al.*, 2005; Searle *et al.*, 2006). Hence, we next analysed the expression of *FD* paralogs in potato by cloning two potato homologs of *AtFD*, namely *StFD* and *StFD-like*. We detected *StFD* expression in the vegetative SAM and in axillary meristems (AMs). During the transition to flowering, *StFD* expression was confined to the IM (Fig. 5C). Afterwards, signals were observed in the IM and AMs, and also in FMs, although they were much weaker in the latter tissue (Fig. 5D, E). Although *AtFD* is expressed in leaf primordia (Abe *et al.*, 2005; Wigge *et al.*, 2005), this was not the case for *StFD*. In addition to its meristem-specific expression, in stages before the transition to flowering we found *StFD-like* in vascular tissue, AMs, and the boundaries between the SAM and young leaf primordia. After the floral induction, its expression domain spread into young vascular tissue in an area just below the SAM. Furthermore, we found signals in IMs and the flower anlagen (Fig. 5I, J).

## Discussion

Due to the importance of potato as a staple crop, determining the regulatory components and the mechanisms of tuber induction are of great interest in order to find candidates that can be targeted for crop improvement. Research on the control of flowering time in potato is increasing, as true potato seeds are becoming important for future breeding endeavours (e.g. Solynta hybrid potato seeds; Lindhout *et al.*, 2018). To study developmental transitions on a detailed molecular level, it is crucial to know about their timing, as this differs for each species with respect to specific growth conditions. With the aim of establishing uniform plant development and synchronized growth starting with *in vitro* cultures, we developed a stringent three-step protocol to study flowering time and tuberization in *S. tuberosum* ssp. *andigena* (Fig. 1). We determined that, similar to Arabidopsis, *andigena* is a facultative LD plant in terms of flowering as we observed that the floral transition occurred much earlier under LD conditions (15–16 DAT) compared to SD conditions (40–50 DAT) (Supplementary Fig. S3). One reason for this delay of the floral transition in SD may have been repression by *StSP6A* in SD conditions, as suggested by Plantenga *et al*. (2018).

Flowering in potato involves complex morphological processes at the SAM. First, mitotic activity, and hence the cell division rate, increases similar to other plant species (Gifford and Tepper, 1961; Steeves *et al.*, 1969; Bodson, 1975; Marc and Palmer, 1982; Jacqmard *et al.*, 2003). We found that potato vegetative SAMs could be clearly distinguished from meristems shortly before or during the transition to flowering (Fig. 2). The SAM underwent high rates of cell division, resulting in a dome-shaped structure. The concomitant cleavage of this transition meristem produced two independent inflorescence meristems. We considered this as the first morphological sign of the reproductive switch and therefore used it as a morphological determinant of the onset of flowering. Concomitant cleavages of both IMs lead to the formation of a double-scorpioid cyme, which is a typical inflorescence architecture in most potato species (Danert ,1957). We used RNA *in situ* hybridization to screen for floral marker genes and their expression patterns during the floral transition. We identified *StAN* and *StWOX9* as suitable markers to distinguish the inflorescence meristem from a flower meristem in a reproductive apex (Fig. 3). Both genes control inflorescence architecture and are sequentially expressed during the formation of flowers in tomato (Lippman *et al.*, 2008). While *StWOX9* was specifically expressed in the inflorescence meristem, *StAN* was expressed in flower organs only, resembling the spatiotemporal expression pattern described for *SlAN* and *SlWOX9* (Lippman *et al.*, 2008). Solanaceae species share common inflorescence architectures, with flowering marking the end of the main shoot growth and axillary meristems continuing the vegetative aerial growth (sympodial growth habit); therefore, *StWOX9* and *StAN* are likely to be involved in the regulation of a developmental program for organizing inflorescence patterning in potato, similar to that of tomato and other members of the Solanaceae (Lippman *et al.*, 2008). Interestingly, this mechanism utilizes conserved components that regulate inflorescence and flower development in other plant species such as Arabidopsis, for example *StAN*, the homolog of Arabidopsis *UNUSUAL FLORAL ORGANS*, or StWOX9, the homolog of *AtSTIMPY* (Ingram *et al.*, 1995; Hofer *et al.*, 1997; Taylor *et al.*, 2001; Zhang *et al.*, 2003; Ikeda *et al.*, 2007; Wu *et al.*, 2007). Our results demonstrate that *StAN* and *StWOX9* can be used as genetic markers in potato for the IM and FM, respectively.

LFY is a plant-specific transcription factor that is found in all land plants and controls general developmental processes in basal plants through to flowering in angiosperms (Maizel *et al.*, 2005). We found that the potato *LFY* homolog, *StLFY*, was expressed throughout development from an early stage (Fig. 4), especially in what seemed like actively growing tissues, and probably correlated with a high number of cell divisions. Localization to growing tissue was supported by the fact that we did not detect signals of *StLFY* expression in the presumptive cleavage site between the inflorescence meristem and the developing flower meristems, which is supposed to be an area of relatively little cell cycle activity (Hart and Hannapel, 2002). It is notable that among the ancestral functions of LFY are the regulation of cell division, expansion, and arrangement (Moyroud *et al.*, 2010). Therefore, we postulate that *StLFY* in potato plays a more general growth-controlling role, whilst not excluding a function during the floral transition via regulation of the determination of cell fate. Interestingly, the expression pattern of the *LFY* homolog *ABERRANT LEAF AND FLOWER* (*ALF*) in petunia (*P. hybrida*), also a member of the Solanaceae, which generates a cymose inflorescence (Souer *et al.*, 2008), is consistent with what we observed in potato. *ALF* is highly expressed in emerging leaf primordia during the vegetative growth phase, and is later expressed in the inflorescence and flower meristems (Souer *et al.*, 1998). It is also of interest that *LFY* expression in Arabidopsis cannot be found in the inflorescence meristem (indeterminate), but instead is found in tissues of determinate organs. The general presence of *LFY* in determinate organs across all plants (including potato, as determined in this study) supports the theory that it controls determinate growth, whereas indeterminate growth is the result of lack of *LFY* expression. Changes of the spatiotemporal expression of vegetative or reproductive identity genes are suggested to have shaped inflorescence architecture during evolution (Prusinkiewicz *et al.*, 2007).

Our study identified *StMC*, the homolog of *AtAP1*, as a marker gene for the floral transition in potato. We found that *StMC* was expressed shortly before the SAM underwent the transition to flowering (Fig. 4). It was highly up-regulated in the presumptive inflorescence meristem, i.e. before the initial cleavage of the meristem (C1). At later stages, *StMC* was highly expressed in the inflorescence meristem and flower organ primordia. In contrast, *AtAP1* expression is activated in lateral flower meristems but decreases in the apical meristem (Mandel *et al.*, 1992; Mandel and Yanofsky, 1995; Blázquez *et al*., 1997; Schmid *et al.*, 2005). The tomato homolog, *SlMC*, has been found to be expressed in inflorescence and flower meristems and is associated with the floral transition (Yuste-Lisbona *et al.*, 2016), suggesting that its role might be conserved in other Solanaceae. Although more work is necessary to determine its role during the transition to flowering in potato, we have identified *StMC* as a suitable marker gene to discriminate between the vegetative and the reproductive stages, given that it was not expressed during the vegetative growth period and was only strongly up-regulated when flowering was induced.

The MADS-box gene family appears to have undergone several gene duplication events (Theissen *et al.*, 2000), which is why functional divergence between different plant species seems likely. Among the MADS-box genes, *AtSOC1* has been shown to integrate signals from different regulatory pathways, such as those of the photoperiod, the ambient temperature, and the autonomous pathway, in order to induce flowering in Arabidopsis (Borner *et al.*, 2000; Lee *et al.*, 2000; Samach *et al.*, 2000). *AtSOC1* mRNA cannot be detected in the Arabidopsis SAM during vegetative development, and is only weakly expressed in leaves. However, following floral induction, it significantly increases in the SAM and is slightly down-regulated in the IM (Olas *et al.*, 2019). AtSOC1, together with its homolog AGAMOUS-LIKE 24 (AtAGL24), promotes the expression of *AtLFY* and *SQUAMOSA PROMOTER BINDING PROTEIN-LIKE3-5* (*AtSPL3-5*) (Lee *et al.*, 2008; Liu *et al.*, 2008; Jung *et al.*, 2012). Interestingly, other MADS-box genes in Arabidopsis, such as *AtAGL42*, *AtAGL71* and *AtAGL72*, are expressed in the vegetative SAM and during the floral transition, where they play a role in the regulation of flowering time (Dorca-Fornell *et al.*, 2011). A possible role of *SOC1* homologs in the regulation of flowering time in Solanaceae species has been reported for *SOC1-like* genes in petunia, where the MADS box gene *UNSHAVEN* (*UNS*) is found to be expressed in vegetative tissues and is down-regulated upon the floral transition and formation of flower meristems (Ferrario *et al.*, 2004). Despite this expression pattern, *UNS* accelerates flowering when overexpressed in petunia and Arabidopsis, and UNS is able to translocate to the nucleus by interacting with *MADS11-like* (homolog of *AtAGL24*; Ferrario *et al.*, 2004), as also shown for AtSOC1 (Lee *et al.*, 2008), suggesting that the biological function of SOC1 might be conserved in plants. Ma *et al.* (2011) showed that overexpression of an additional *SOC1-like* gene from petunia (*FBP21*) led to early flowering in tobacco plants and resulted in an up-regulation of the tobacco *LFY* and *AP1* homologs. In potato, *StSOC1* was highly expressed at the SAM during the vegetative phase, and decreased at later stages after the floral induction (Fig. 4). This pattern resembles the expression of the floral integrator *UNS* in petunia; therefore, we believe that *StSOC1* may act as a floral integrator in potato. Although *StSOC1-like* was only weakly expressed at the apex, the expression level alone cannot exclude the possibility that *StSOC1-like* plays a role during the floral transition.

In some plant species, such as *S. lycopersicum* and *O. sativa*, the transition to flowering is regulated by a protein complex comprised of FT and FD (Pnueli *et al.*, 2001; Taoka *et al.*, 2011). In our study, the expression patterns of both *StFD* candidates (*StFD* and *StFD-like*; Fig. 5) at the potato shoot apex indicated that StFD might fulfil a similar role by interacting in the SAM with StSP3D (the florigen in potato) in order to activate the expression of downstream target genes, eventually inducing flowering. Similar to *AtFD* (Wigge *et al.*, 2005), *StFD* transcripts were specifically found in meristems throughout the transition from the vegetative to reproductive stage. To date, little is known about the role of *FD* genes in potato; however, *StFD-like* has been implicated in the control of tuber formation, and both StFD and StFD-like have been shown to interact with StSP6A via 14-3-3 proteins, supporting the idea that FD–FT protein complexes might regulate developmental transitions in general (Teo *et al.*, 2017). In summary, our expression analyses indicate a role of StFDs at the shoot apex in potato, and it seems likely to contribute in the regulation of flowering time.

We strongly believe that knowledge gained in Arabidopsis can serve as a basis to understand developmental transitions and the underlying regulatory networks in *S. tuberosum*. Given the fact that flowering and tuberization employ similar pathway components (Rodríguez-Falcón *et al*., 2006), it will be interesting to investigate the genes identified in this study during the stolon-to-tuber transition. In particular, it will be important to address the SD-dependent regulation of tuberization in ancestral genotypes such as *andigena* in comparison to commercial cultivars, which have lost this trait through breeding for tuberization under longer days (Kloosterman *et al.*, 2013). Interestingly, we have recently found an *andigena*-specific tolerance to limited nitrogen conditions in terms of tuber formation, indicating the rich trait potential of ancestral genotypes for new breeding opportunities (Van Dingenen *et al.*, 2019). Clearly, more work will be necessary to disentangle and identify the regulatory pathways controlling flowering and tuberization, but future potato breeding will eventually benefit from this knowledge as it will aid in directing the addition or modification of specific traits, in contrast to conventional breeding strategies.

## Supplementary data

Supplementary data are available at *JXB* online.

Supplementary data S1. Determination of stolon-to-tuber development in *S. tuberosum* ssp. *andigena*.

Fig. S1. Experimental design to morenitor stolon initiation under LDs and tuberization in response to a LD/SD shift in potato.

Fig. S2. Phenotype of *S. tuberosum* ssp. *andigena* under LD conditions.

Fig. S3. Flowering time of *S. tuberosum* ssp. *andigena* plants grown in LD and SD conditions.

Fig. S4. Protein alignments for StANANTHA and selected homologs.

Fig. S5. Protein alignments for StWOX9 and selected homologs.

Fig. S6. Protein alignments for StLEAFY and selected homologs.

Fig. S7. Protein alignments for StMACROCALYX and selected homologs.

Fig. S8. Protein alignments for StSOC1 and selected homologs.

Fig. S9. Protein alignments for StFD and selected homologs.

Fig. S10. Hybridization of sense probes as controls for RNA *in situ* hybridizations in this study.

Table S1. Gene IDs and sequences of oligonucleotides used in this study

erz484_suppl_Supplementary_Data

erz484_suppl_Supplementary_Figures_S1_S10_and_Table_S1

## Abbreviations

AM: axillary meristem
C1: initial cleavage of the SAM
DAT: days after transfer
DM: dome-shaped meristem
FM: flower meristem
IM: inflorescence meristem
LD: long days
LP: leaf primordia
SAM: shoot apical meristem
SD: short days
VM: vegetative meristem
