# Supplementary material for: Flowering time and the identification of floral marker genes in Solanum tuberosum ssp. andigena
Source: J Exp Bot. 2019 Oct 28;71(3):986–96. doi: 10.1093/jxb/erz484 (PMC6977542; doi:10.1093/jxb/erz484)
Supplement: erz484_suppl_Supplementary_Data [file erz484_suppl_supplementary_data.docx]

**Supplementary data**

**Fig. S1** Experimental design to monitor stolon initiation (LD) and tuberization in a LD/SD shift in potato.

**Fig. S2** Phenotype of *S. tuberosum* ssp. *andigena* under LD conditions.

**Fig. S3** Flowering time of *S. tuberosum* ssp. *andigena* plants grown in LD and SD conditions.

**Fig. S4** Protein alignments for StANANTHA.

**Fig. S5** Protein alignments for StWOX9.

**Fig. S6** Protein alignments for StLEAFY.

**Fig. S7** Protein alignments for StMACROCALYX.

**Fig. S8** Protein alignments for StSOC1.

**Fig. S9** Protein alignments for StFD.

**Fig. S10** Sense probes as control for RNA *in situ* hybridizations in this study.

**Table S1** Gene IDs and sequences of oligonucleotides used in this study.
