# Supplementary material for: Flowering time and the identification of floral marker genes in Solanum tuberosum ssp. andigena
Source: J Exp Bot. 2019 Oct 28;71(3):986–96. doi: 10.1093/jxb/erz484 (PMC6977542; doi:10.1093/jxb/erz484)
Supplement: erz484_suppl_Supplementary_Figures_S1_S10_and_Table_S1 [file erz484_suppl_supplementary_figures_s1_s10_and_table_s1.pdf]

Supplementary File

Stolon to tuber development in *S. tuberosum* ssp. *andigena*

Alike flowering, tuberization is a developmental process involving a variety of morphological changes such as the swelling of the stolon tip involving a change in the cell division plane and an increase in radial cell expansion (Xu *et al.,* 1998, Vreugdenhil *et al.,* 1999). Long day to short day (LD/SD) shift experiments were performed in order to characterize stolon initiation time in LD conditions and stolon to tuber development in SD conditions (see Figure S1 A and C for growth regime). For this purpose, stolon initiation of LD grown wild-type plants was monitored at 11, 14, 17, 20, 23 and 26 DAT (Figure S1 A). At 11 DAT, no stolons were initiated from below ground nodes. After 14 DAT the first stolons were visible, however with a maximum of two stolons per plant and an approximate length of only 1 cm. During the following time points stolon initiation and thereby the number of stolons gradually increased to 2-5 main stolons per plant which were growing between 4-6 cm in length (Figure S1 B). As soon as the initiated stolons perceived light through a loosely covering soil layer, they bend upwards towards the light and started growing to eventually produce a new vegetative shoot. In order to prevent the loss of these stolons, an additional layer of soil was added to the plants after 14 DAT.

For a sufficient number and length of stolons per plant *S. tuberosum* ssp. *andigena* plants where grown for at least 27 days in LD conditions. These plants were then subjected to SD photoperiod for another 18 days to induce photoperiod-dependent tuberization (Figure S1 C). During the 18 days in SD, plants were monitored every day for different developmental stolon and tuber stages. For sampling we referred to a stage-dependent classification (Figure S1 E) as described by Kloosterman and colleagues (Kloosterman *et al.,* 2005). During the first 4 days after the shift (DAS), stolons of stage 1-3 were observed, while at 5 DAS we monitored

stages 3-5. At later harvesting time points (8 DAS and 10 DAS) tubers were grown up to 2-3 cm (stages 7-8). It is important to mention that besides the primary stolons which are initiated during the first three weeks, secondary (lateral) stolons arise from the axils of primary stolons. This branching of stolons leads to various developmental stages on one plant also at later time points after tuber induction (Figure S1 D), indicating that a synchronized tuberization of all stolon tips is not possible. None of the plants grown in the LD experiments in which plants were not shifted to short days produced tubers, confirming the strict SD-dependent tuberization in *S. tuberosum* ssp. *andigena* under our growth conditions.

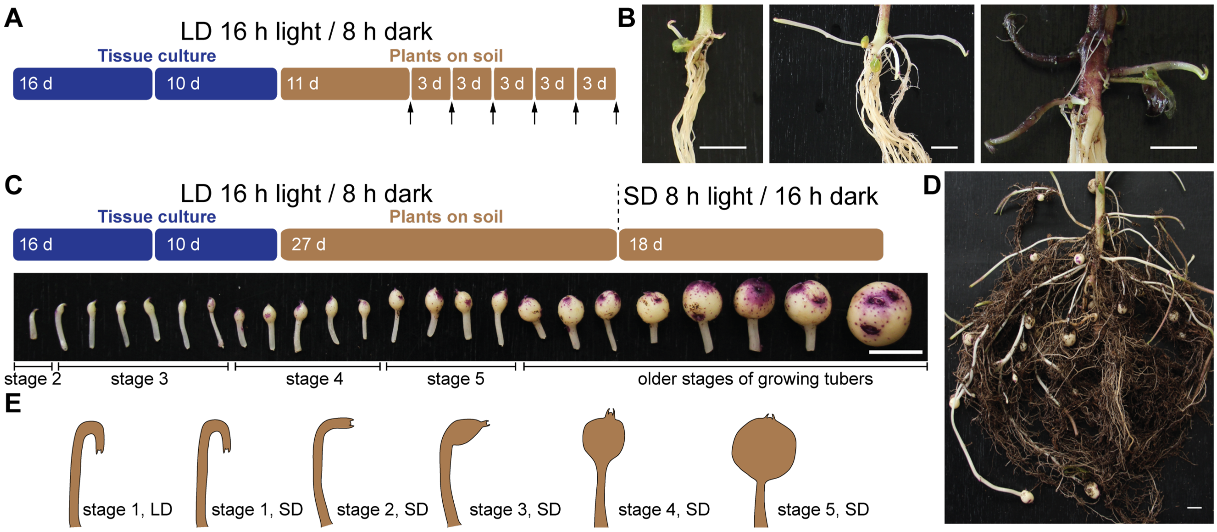

Fig. S1 Experimental design to monitor stolon initiation (LD) and tuberization in a LD/SD shift in potato. (**A**) In order to analyze the time point of stolon initiation and growth in LD conditions plants were grown for 11 DAT in LD and harvested every three days to analyze below ground stolon formation. (**B**) Stolons of representative plants at 14, 20 and 26 DAT. (**C**) Stolon to tuber development was monitored in a second experiment in which tuberization was induced by a shift to SD conditions and plants were grown for 18 additional days in SD. The picture in (**C**) depicts a stolon to tuber developmental series of stolons and tubers derived from three different Andigena plants 18 DAS (days after shift). Developmental

stages were classified according to Kloosterman and colleagues (Kloosterman *et al.,* 2005). (**D**) Root ball with stolons and tubers of one representative plant after 18 DAS. (**E**) Schematic representation of the six developmental stages of tuber development: stage 1 LD, non-induced stolon with a closed apical hook; stage 1 SD, induced stolon, closed hook; stage 2 SD, induced stolon with an opened apical hook; stage 3 SD, induced stolon, open hook and swollen apical region; stage 4 SD, swollen stolon with an approximate size of 0.5 cm, the apical tip of the stolon is still clearly visible; stage 5 SD, young tuber where the tip of the stolon is not clearly distinguishable any longer. Scale bars: 1cm.

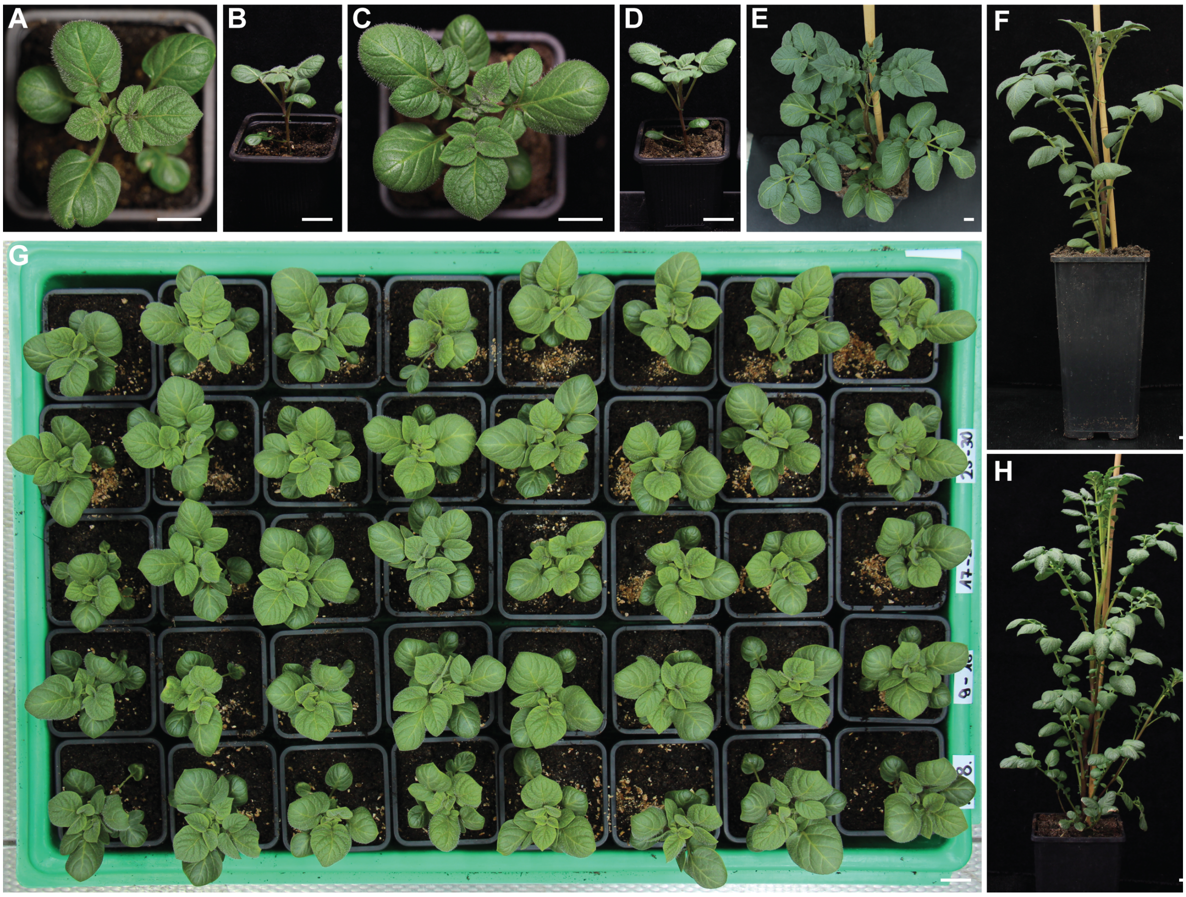

Fig. S2 Plant development of *S. tuberosum* ssp. *andigena* under LD conditions. Wild-type plants in tissue culture (not shown) were grown using the three-step protocol described in this work. Plants were transferred to soil into small pots and grown for 14 days, followed by re-potting into bigger pots allowing stolon and tuber formation (tuber formation only when plants were shifted to SD). Growth conditions: 16 h light, 8 h dark; 22 °C, 60% humidity, 300-400 µmol m^{-2} s^{-1}. Pictures were taken 11 (**A**, **B**, **G**), 14 (**C**, **D**), 18 (**E**, **F**), and 39 DAT (**H**). Scale bar: 2 cm.

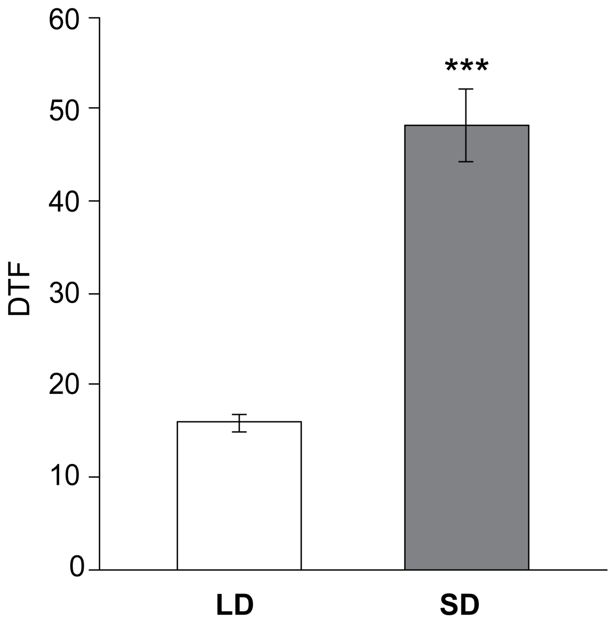

Fig. S3 Flowering time of *S. tuberosum* ssp. *andigena* plants grown in LD and SD conditions. Flowering time was analyzed using a stereo microscope, and flowering time was scored based on morphological traits of the meristem. Days to flowering (DTF) is represented as the average day after the transfer to soil (DAT) when plants where flowering. Error bars indicate standard deviation of the mean (SD n=6, LD n=70). Two-tailed Student's t-test: ***$p<0.001$.

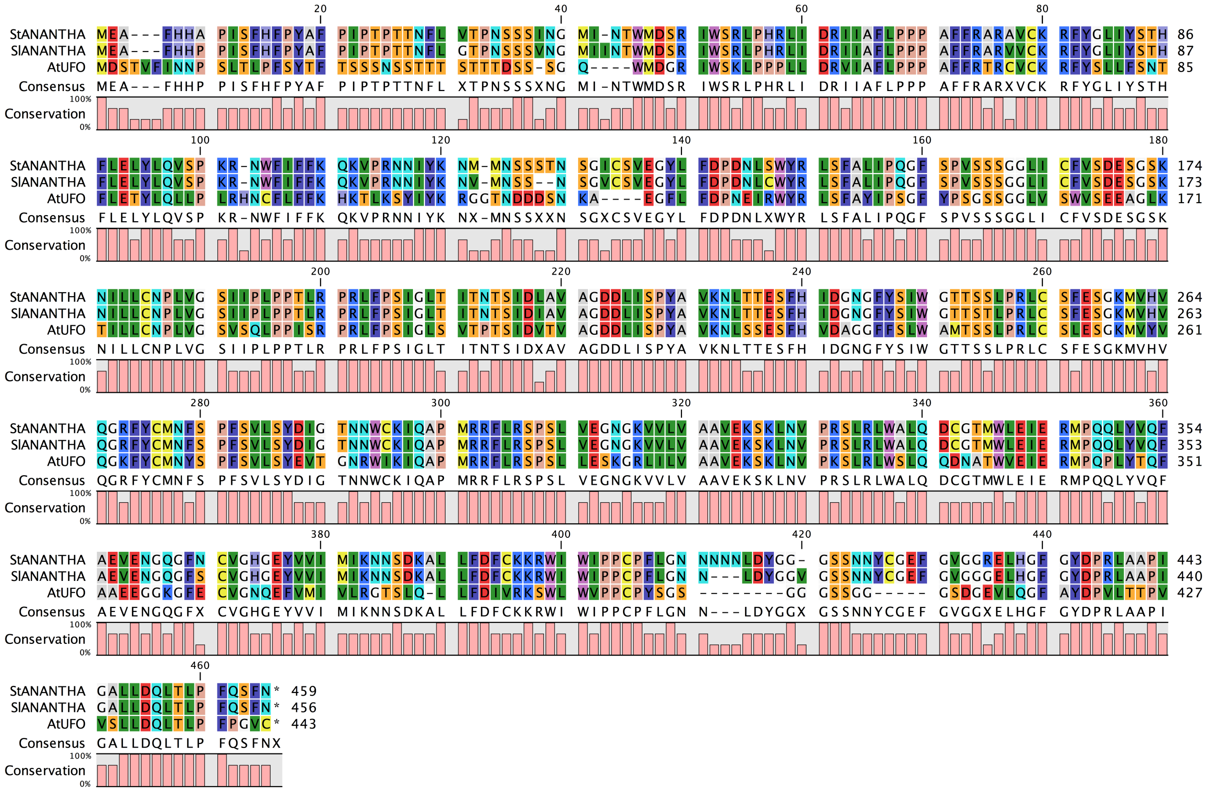

Fig. S4 Protein alignment of ANANTHA from *S. lycopersicum* and *S. tuberosum* and its homolog AtUFO from *A. thaliana*. Whole protein sequences were obtained from the SpudDB Potato Genomics Resource and the NCBI GenBank and where aligned using CLCSequenceViewer7 (Qiagen Bioinformatics). (SlANANTHA – Solyc02g081670; AtUFO – At1g30950).

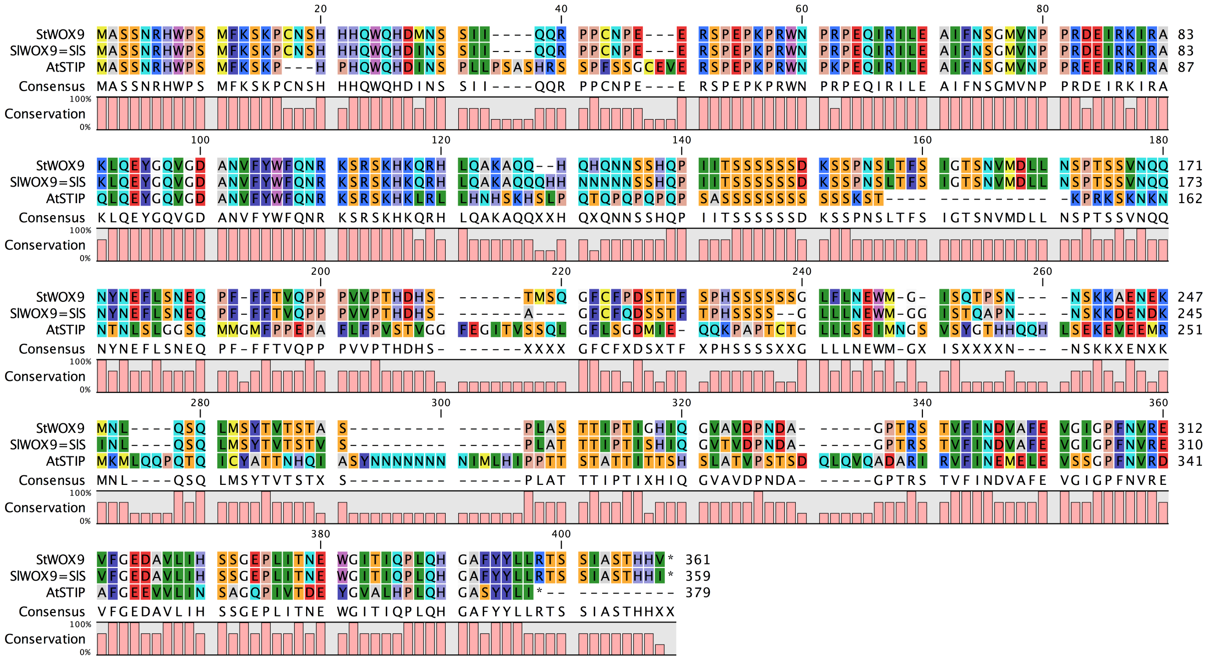

Fig. S5 Protein alignment of WOX9 from *S. lycopersicum* and *S. tuberosum* and its homolog AtSTIP from *A. thaliana*. Whole protein sequences were obtained from the SpudDB Potato Genomics Resource and the NCBI GenBank and where aligned using CLCSequenceViewer7 (Qiagen Bioinformatics). (SlWOX9 – Solyc02g077390; AtSTIP – At2g33880).

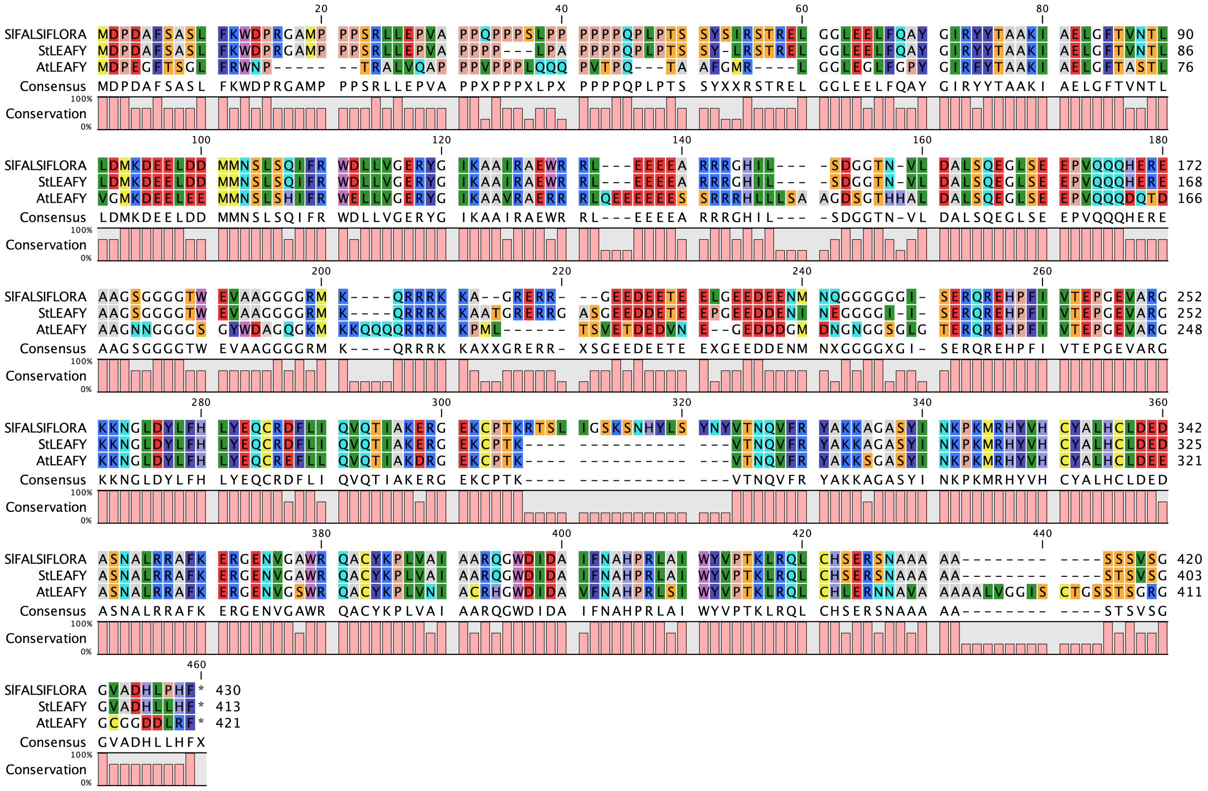

Fig. S6 Protein alignment of LEAFY from *S. lycopersicum*, *S. tuberosum*, and *A. thaliana*. Whole protein sequences were obtained from the SpudDB Potato Genomics Resource and the NCBI GenBank and where aligned using CLCSequenceViewer7 (Qiagen Bioinformatics). (SlFALSIFLORA – Solyc03g118160; AtLEAFY – At5g61850).

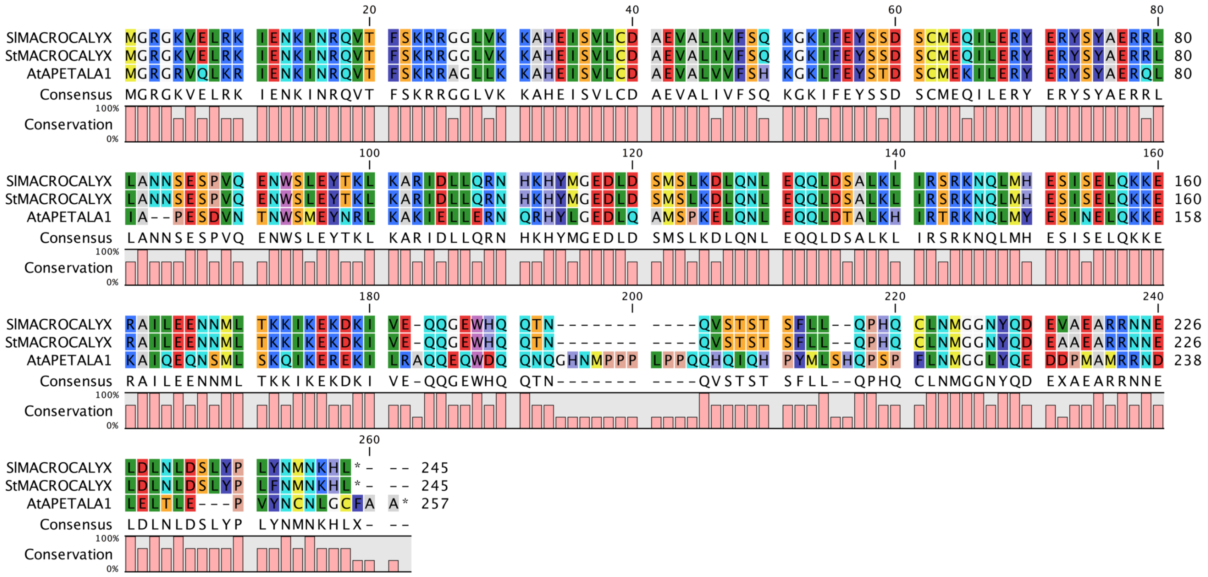

Fig. S7 Protein alignment of MACROCALYX from *S. lycopersicum* and *S. tuberosum*, and its homolog AtAPETALA1 from *A. thaliana*. Whole protein sequences were obtained from the SpudDB Potato Genomics Resource and the NCBI GenBank and where aligned using CLCSequenceViewer7 (Qiagen Bioinformatics). (SlMACROCALYX – Solyc05g056620; AtAPETALA1 – At1g69120).

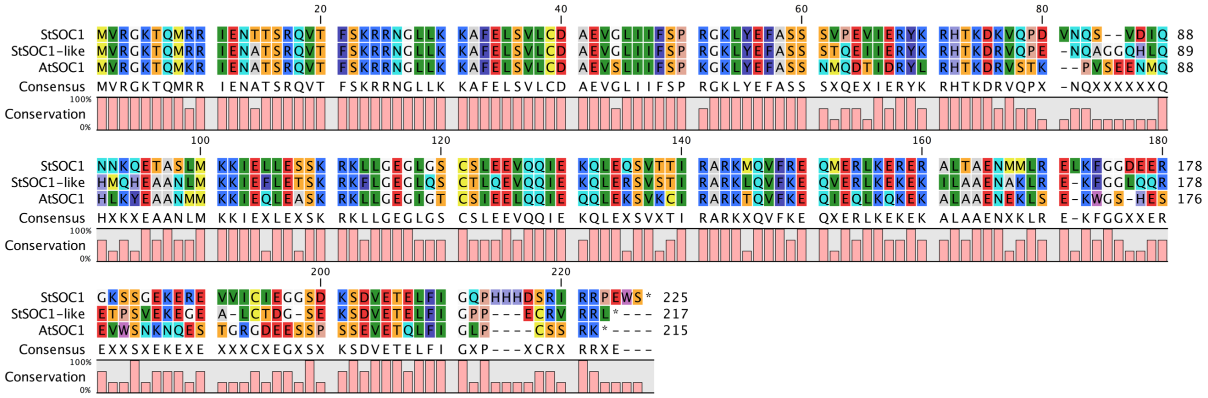

Fig. S8 Protein alignment of SOC1 from *S. tuberosum* and *A. thaliana*. Whole protein sequences were obtained from the SpudDB Potato Genomics Resource and the NCBI GenBank and where aligned using CLCSequenceViewer7 (Qiagen Bioinformatics). (AtSOC1 – At2g45660).

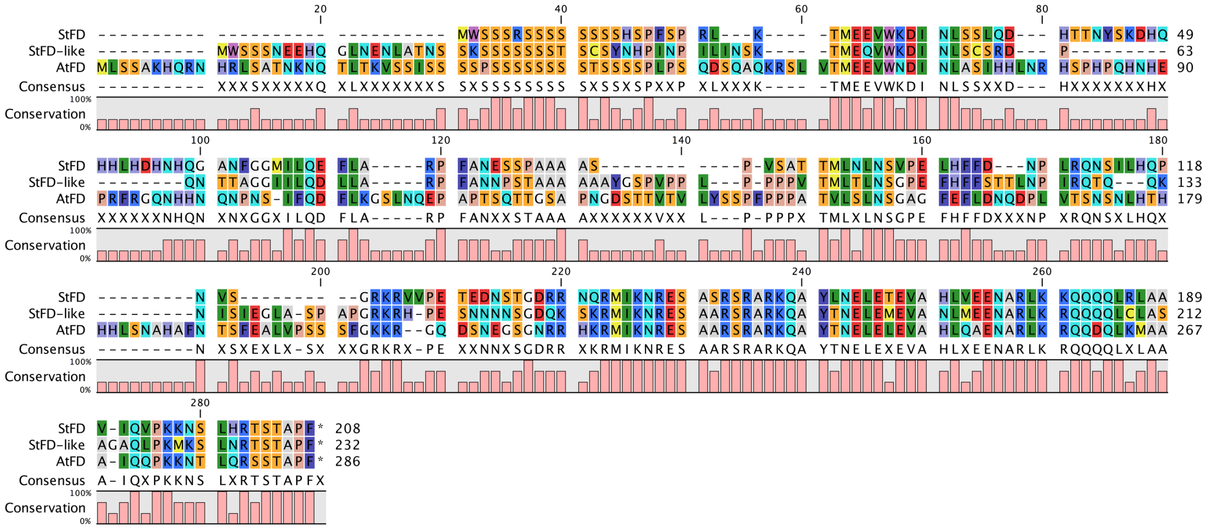

Fig. S9 Protein alignment of FD from *S. tuberosum* and *A. thaliana*. Whole protein sequences were obtained from the SpudDB Potato Genomics Resource and the NCBI GenBank and where aligned using CLCSequenceViewer7 (Qiagen Bioinformatics). (AtFD – At4g35900).

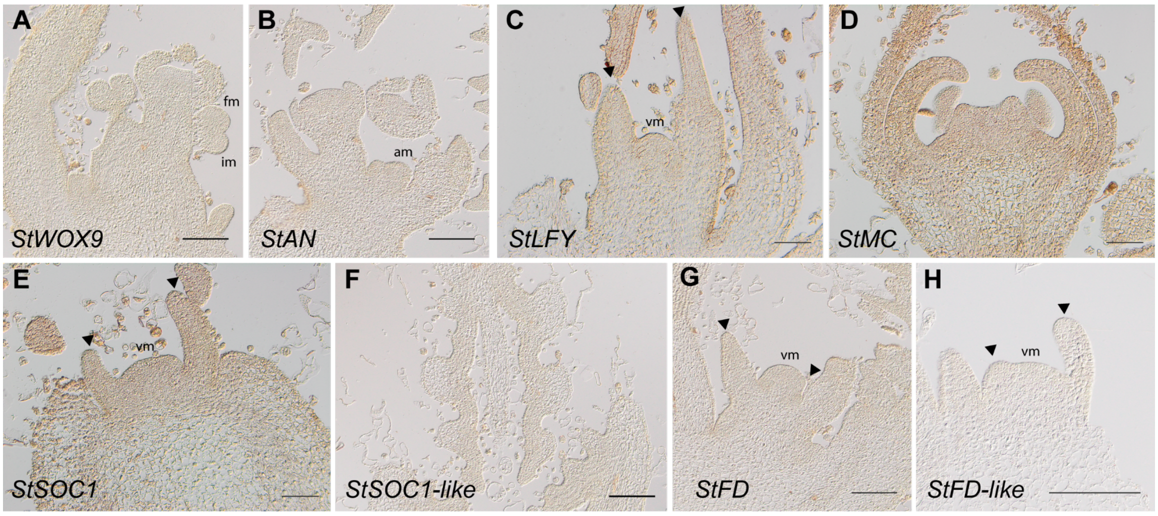

Fig. S10 Sense probes as control for RNA *in situ* hybridizations in this study. Hybridization was done using longitudinal sections of the shoot apex of plants grown in LD conditions. (**A**) Sense probe of *StWOX9*. (**B**) Sense probe of *StAN*. (**C**) *StLFY* and (**D**) *StMC* sense probes. (**E**) *StSOC1* and (**F**) *StSOC1-like* sense probes. (**G**) *StFD* and (**H**) *StFD-like* sense probes. No signals were observed for sense probes. vm: vegetative meristem; im: inflorescence meristem; fm: flower meristem; am: axillary meristem; arrowheads indicate leaf primordia. Scale bars: 100 µm.

Table S1: Gene IDs and sequences of oligonucleotides used in this study.

Gene	Gene ID	Primer sequences
StANANTHA	PGSC0003DMT400045739	ATGGAAGCTTTTCATCATGCC<br>TCAGTTGAATGACTGAAAGGG
StWOX9	PGSC0003DMT400027850	ATGGCTTCATCAAATAGACAC<br>TTATACATGATGAGTCGATGC
StLFY	PGSC0003DMT400036749	ATGGACCCAGATGCTTTCTC<br>TTAGAAATGCAGCAGGTGAT
StMC	PGSC0003DMT400072888	ATGGGAAGAGGAAAAGTTG<br>TCATAGATGTTTATTCATGTTG
StSOC1	PGSC0003DMT400000028	ATGGTGAGAGGGAAAACACAG<br>TCAAGACCACTCAGGACGC
StSOC1-like	PGSC0003DMT400000030	ATGGTGAGAGGGAAAACACAG<br>TTAGAGACGCCTTACTCTGC
StFD	PGSC0003DMT400009393	ATGTGGTCATCAAGCAGGTC<br>TCAAAATGGAGCGGTTGACG
StFD-like	PGSC0003DMT400061403	ATGTGGTCATCAAGTAATG<br>TCAAAATGGGGCCGTTGATG

Supplementary References

Kloosterman B, Vorst O, Hall RD, Visser RG, Bachem CW. 2005. Tuber on a chip: differential gene expression during potato tuber development. Plant biotechnology journal. **5**, 505-19.

Vreugdenhil D, Xu X, Jung CS, van Lammeren AAM, Ewing EE. 1999. Initial Anatomical Changes Associated with Tuber Formation on Single-node Potato (Solanum tuberosum L.) Cuttings: A Re-evaluation. Annals of Botany. **5**, 675-80.

Xu X, Vreugdenhil D, Lammeren AAMv. 1998. Cell division and cell enlargement during potato tuber formation. Journal of experimental botany. **320**, 573-82.
